# Effect of the HPV vaccination programme on incidence of cervical cancer and grade 3 cervical intraepithelial neoplasia by socioeconomic deprivation in England: population based observational study

**DOI:** 10.1136/bmj-2023-077341

**Published:** 2024-05-15

**Authors:** Milena Falcaro, Kate Soldan, Busani Ndlela, Peter Sasieni

**Affiliations:** 1 Centre for Cancer Screening, Prevention and Early Diagnosis, Wolfson Institute of Population Health, Queen Mary University of London, London EC1M 6BQ, UK; 2 Blood Safety, Hepatitis, Sexually Transmitted Infections and HIV Division, UK Health Security Agency (UKHSA), London, UK; 3 National Disease Registration Service (NDRS), NHS England, London, UK

## Abstract

**Objectives:**

To replicate previous analyses on the effectiveness of the English human papillomavirus (HPV) vaccination programme on incidence of cervical cancer and grade 3 cervical intraepithelial neoplasia (CIN3) using 12 additional months of follow-up, and to investigate effectiveness across levels of socioeconomic deprivation.

**Design:**

Observational study.

**Setting:**

England, UK.

**Participants:**

Women aged 20-64 years resident in England between January 2006 and June 2020 including 29 968 with a diagnosis of cervical cancer and 335 228 with a diagnosis of CIN3. In England, HPV vaccination was introduced nationally in 2008 and was offered routinely to girls aged 12-13 years, with catch-up campaigns during 2008-10 targeting older teenagers aged <19 years.

**Main outcome measures:**

Incidence of invasive cervical cancer and CIN3.

**Results:**

In England, 29 968 women aged 20-64 years received a diagnosis of cervical cancer and 335 228 a diagnosis of CIN3 between 1 January 2006 and 30 June 2020. In the birth cohort of women offered vaccination routinely at age 12-13 years, adjusted age standardised incidence rates of cervical cancer and CIN3 in the additional 12 months of follow-up (1 July 2019 to 30 June 2020) were, respectively, 83.9% (95% confidence interval (CI) 63.8% to 92.8%) and 94.3% (92.6% to 95.7%) lower than in the reference cohort of women who were never offered HPV vaccination. By mid-2020, HPV vaccination had prevented an estimated 687 (95% CI 556 to 819) cervical cancers and 23 192 (22 163 to 24 220) CIN3s. The highest rates remained among women living in the most deprived areas, but the HPV vaccination programme had a large effect in all five levels of deprivation. In women offered catch-up vaccination, CIN3 rates decreased more in those from the least deprived areas than from the most deprived areas (reductions of 40.6% *v* 29.6% and 72.8% *v* 67.7% for women offered vaccination at age 16-18 and 14-16, respectively). The strong downward gradient in cervical cancer incidence from high to low deprivation in the reference unvaccinated group was no longer present among those offered the vaccine.

**Conclusions:**

The high effectiveness of the national HPV vaccination programme previously seen in England continued during the additional 12 months of follow-up. HPV vaccination was associated with a substantially reduced incidence of cervical cancer and CIN3 across all five deprivation groups, especially in women offered routine vaccination.

## Introduction

Human papillomavirus (HPV) comprises a family of viruses, a subset of which are responsible for virtually all cervical and some anogenital and oropharyngeal cancers.^1^ More than 100 countries worldwide have introduced prophylactic HPV vaccination as part of routine immunisation schedules.^2^ One important outcome yet to be reported is whether vaccination has reduced or increased the inequalities seen for cervical disease in the UK and elsewhere.

In England, the national HPV vaccination programme started in 2008 using the bivalent Cervarix vaccine to prevent infections due to HPV types 16 and 18, which are estimated to cause around 80% of all cervical cancers in the UK.^3^ Vaccination was offered routinely to 12-13 year old (school year 8) girls and as part of a catch-up campaign to those aged <19 years.^4^ In September 2012 the programme switched to the quadrivalent vaccine (Gardasil), which additionally protects against HPV types 6 and 11 (responsible for genital warts), and in 2019 the programme was extended to 12-13 year old boys. Those who are eligible but not vaccinated can receive the vaccine free of charge from their general practitioner until their 25th birthday.^5^

The introduction and implementation of HPV immunisation in this way means that noticeable discontinuities exist in the proportion of women vaccinated by date of birth, enabling a rigorous evaluation of the effectiveness of the programme.^6^ For example, women born in August 1990 are unlikely to have received HPV vaccination, whereas among those born in the year from 1 September 1990 nearly 70% have received at least one dose of the vaccine.

Findings on the early effect of national HPV vaccination programmes have been encouraging. A wealth of real world evidence for the effect of vaccination on HPV prevalence exists^7^ ^8^ ^9^ ^10^ ^11^ and evidence is growing for its effectiveness in reducing high grade cervical intraepithelial neoplasia (CIN)^12^ ^13^ ^14^ ^15^ and cervical cancer in vaccinated women.^14^ ^16^ ^17^ ^18^ ^19^ For instance, we found that in England rates of grade 3 CIN (CIN3) and of cervical cancer were greatly reduced among those who were offered HPV vaccination, and that the magnitude of the reduction was greatest in the cohorts with the highest uptake and younger age at vaccination.^14^ We estimated that by mid-2019 the immunisation programme had prevented cervical cancer in nearly 450 women and CIN3 in around 17 000 women.

Along with preventing ill health, a key aim of the NHS is to reduce health inequalities.^20^ To this end, we investigated whether the effect of immunisation against HPV has resulted in a reduction in inequalities in cervical disease or a widening. Concern has been expressed that if the uptake of HPV vaccination is lower in those at greatest risk of cervical cancer, as has been seen in the US,^21^ this could accentuate health inequalities. One study found that the introduction of HPV immunisation in England might initially have increased inequities in HPV related cancer incidence among ethnic minority groups because of the differential effect of herd protection in subpopulations with dissimilar vaccination coverage.^22^ Previous studies have suggested that white people have a higher awareness of HPV and acceptance of the immunisation^23^ and that vaccination uptake is lower in women from ethnic minority groups and more deprived areas.^24^ Using data on HPV vaccination coverage by local area, however, a study found little variation by deprivation score in women offered routine vaccination (83% *v* 86% for most and least deprived areas, respectively) and only a small negative correlation between deprivation and vaccine uptake in those offered catch-up vaccination (47% *v* 53% for most and least deprived areas, respectively).^25^ A full understanding of the effect of HPV vaccination across different socioeconomic groups is complicated by the poor uptake of cervical screening observed among younger women in the most deprived areas, leading to lower rates of screen detected cervical cancer and CIN3 at age 25 years compared with women in less deprived areas.^26^ ^27^

We replicated results from an analysis of population based cancer registry data to evaluate if the high vaccination effectiveness seen previously continued during an additional year of follow-up. The combined data were also used to investigate the effect of the vaccination programme by socioeconomic deprivation.

## Methods

To represent socioeconomic deprivation, we used the index of multiple deprivation, a small area measure based on several domains of deprivation, such as income, employment, and health. The index is determined by using a standard statistical geographical unit, called lower super output area, which divides England into small areas of similar sized populations (on average about 1500 residents, or 650 households).^28^ The lower super output areas are then ranked from the most to the least deprived and divided into five equal groups. The first and fifth groups correspond to the 20% most deprived and 20% least deprived lower super output areas in England, respectively.

We retrieved the records of all women aged 20-64 years resident in England with a diagnosis of invasive cervical cancer (ICD-10 (international classification of diseases, 10th revision) code C53) or CIN3 (ICD-10 code D06) between 1 January 2006 and 30 June 2020. These records are stored in the database managed by NHS England’s National Disease Registration Service,^29^ and for each patient included information on index of multiple deprivation derived from the patient’s home postcode at the time of diagnosis. To convert these counts into rates, we used mid-year estimates of the female population for England by single year of age, calendar year (January 2006 to June 2020), and index of multiple deprivation (five groups). These estimates were retrieved from multiple tables publicly available on the website of the UK’s Office for National Statistics (ONS).^30^ The supplementary material provides more details about the index of multiple deprivation versions used by the National Disease Registration Service and ONS, along with information on how we derived the population estimates required in our statistical analysis.

### Statistical analysis

We separately analysed incidence rates of cervical cancer and CIN3 by using extensions of our previously described age-period-cohort Poisson model.^14^ ^31^ ^32^ Data on women with cancer or CIN3 were aggregated by single month of age, calendar time (period), and date of birth (cohort). We derived the corresponding population risk time by subdividing the mid-year ONS population estimates into one month intervals for age, period, and cohort. For the analysis of the effectiveness by deprivation, we further split both the data on women with cancer or CIN3 and the population estimates by deprivation group (fifths). We then used the population risk time as the denominator for calculating rates (formally, the subdivided population estimates were log transformed and included in the Poisson regression model as an offset). Confidence intervals were computed using robust standard errors.^33^ ^34^

The code for the analysis was written and tested on synthetic data (extending the Simulacrum dataset)^35^ by a statistician (MF) at King’s College London and then run on the real dataset by an analyst (BN) at the National Disease Registration Service.

We started by considering a core model where we included the main effects for age, period, and birth cohort, along with selected age by cohort and age by period interactions (see supplementary table S1). The interaction terms were included to account for variations in screening policy and historical events that affected cervical cancer rates. Specifically, we defined seven birth cohorts to capture differences in the age at first invitation to screening and the school years in which HPV vaccination was offered (see table 1). We added terms for seasonality and for events that may have affected registrations for cervical cancer and CIN3, such as the covid-19 lockdown, the “Jade Goody effect,”^36^ ^37^ and the 2019 cervical screening awareness campaign. In our previous paper,^14^ we used several similar regression models to study the sensitivity of results to the precise way in which we adjusted for potential confounding factors. Because we found that the estimates of the cohort specific incidence rate ratios changed little across the various models, here we report on only a single model adjustment for confounders.

**Table 1:** Characteristics of the birth cohorts

Birth cohort	Date of birth	Age at first invite for screening (years)	Offered vaccination	HPV vaccination			
				Type of campaign	Age (years)	School year	Vaccine coverage (%)*
Cohort 1	≤August 1984	20	No	-	-	-	0
Cohort 2	September 1984-October 1985	20 or 25	No	-	-	-	0
Cohort 3	November 1985-April 1989	25	No	-	-	-	0
Cohort 4	May 1989-August 1990	24.5	No	-	-	-	0
Cohort 5	September 1990-August 1993	24.5	Yes	Catch-up	16-18	12-13	38.9-48.1
Cohort 6	September 1993-August 1995	24.5	Yes	Catch-up	14-16	10-11	70.8-75.7
Cohort 7	September 1995-June 2000	24.5	Yes	Routine	12-13	8	80.9-88.0

HPV=human papillomavirus.

* National annual uptake of full dose, including mop-up vaccinations when data were available.

Using the core model described, we investigated if the high effectiveness of the HPV immunisation programme reported previously^14^ continued during an additional 12 months of follow-up. To do this we split the main effect of each cohort offered vaccination into two subgroup effects depending on whether the data related to the periods 1 January 2006 to 30 June 2019 or 1 July 2019 to 30 June 2020; this approach corresponded to adding three cohort by period interaction terms.

To evaluate the impact of socioeconomic deprivation on incidences of cervical cancer and CIN3, we extended the core model by adding main effects for deprivation and deprivation by cohort interactions. Specifically, we allowed the effect of each deprivation level to vary between unvaccinated women (cohorts 1-4) and those offered vaccination (cohorts 5-7), but we assumed it was otherwise constant within these two groups. We did not include further interactions between deprivation and other covariates as they were not of primary interest in this analysis. Using the fitted Poisson regression models, we made “what if” predictions by changing the value of one or more predictors and by leaving the others as observed. In this way it was possible to compare what happened (factual scenario) with what would have happened under an alternative (counterfactual) scenario.

We also carried out a sensitivity analysis where the effects of these deprivation by cohort interactions were allowed to vary across the three different groups offered vaccination (ie, we used 15 terms instead of five). For cervical cancer, owing to small numbers in cohort 7, we fitted a reduced model where the effects of these interactions were constrained to be the same for cohorts 6 and 7.

All analyses were performed in Stata, version 17.^38^

### Patient and public involvement

Patient and public involvement contributors were not formally involved in this research. We did, however, engage with Cancer Research UK (CRUK), Jo’s Cervical Cancer Trust, and the HPV Coalition on the importance of these analyses and the dissemination of the results. This included taking part in a video produced by ITN Business for World Cancer Day 2023, writing a piece for the 20th anniversary of the creation of CRUK, and engaging with international media about our research findings on the effect of the English HPV vaccination programme. We have also discussed the research and a draft of this paper with individual patients, journalists, and patient and public involvement representatives linked to broader research programmes.

## Results

Table 1 lists the characteristics of the birth cohorts included in the study. We defined the different cohorts so that each cohort is homogeneous in terms of the age women would have been offered HPV vaccination (if at all) and the age at which they would have first been invited for cervical screening. 

Overall, there were 231.1 million women years of observation between 1 January 2006 and 30 June 2020 on women aged 20-64 years in England. During this time, 29 968 women received a diagnosis of invasive cervical cancer and 335 228 a diagnosis of CIN3 (table 2). Observations between 1 July 2019 and 30 June 2020 have not been reported previously. With these additional 12 months of follow-up, there are, in the routine vaccination group (cohort 7), about twice the number of diagnoses compared with the same group in our previous study (we now have 13 *v* 7 previously for cervical cancer, 109 *v* 49 for CIN3; see supplementary table S2).

**Table 2:** Summary statistics of study population

	Invasive cervical cancer	CIN3	Total women years in population (millions)
**Birth cohorts**			
Cohort 1: Invited from age 20 and unvaccinated	25 062	211 501	186.3
Cohort 2: Invited from age 20 or 25 and unvaccinated	1021	21 629	6.2
Cohort 3: Invited from age 25 and unvaccinated	2453	59 881	16.5
Cohort 4: Invited from age 24.5 and unvaccinated	650	18 747	5.1
Cohort 5: Invited from age 24.5 and offered vaccine in school years 12-13 (ages 16-18)	669	19 920	9.1
Cohort 6: Invited from age 24.5 and offered vaccine in school years 10-11 (ages 14-16)	100	3441	4.0
Cohort 7: Not invited before age 24.5 and offered vaccine in school year 8 (ages 12-13)	13	109	3.9
**Age at diagnosis (years)**			
20 to <24.5	337	9954	22.3
24.5 to <26	1609	59 539	8.1
26 to <30	3533	92 568	21.2
30 to <65	24 489	173 167	179.5
**Period of diagnosis**			
January 2006-December 2018	26 826	307 231	206.6
January 2019-September 2019	1722	16 118	12.3
October 2019-June 2020	1420	11 879	12.3
**Deprivation (fifths)**			
1st (most deprived 20%)	8229	83 680	46.9
2nd	6589	73 982	48.2
3rd	5639	66 311	46.8
4th	5091	59 514	45.4
5th (least deprived 20%)	4420	51 741	44.0

CIN3=grade 3 cervical intraepithelial neoplasia.

Our previously published findings on the effect of the national HPV vaccination were largely confirmed with the new data (table 3, also see supplementary table S3). The analysis showed that the previously observed low rates of disease and the estimated high effectiveness of the immunisation programme continued during the additional 12 months of follow-up (diagnoses in July 2019 to June 2020) among women born since 1 September 1990. In particular, the estimated effects of vaccination for that later period in cohort 7 (those born since 1 September 1995) imply a reduction in incidence of 83.9% (95% confidence interval (CI) 63.8% to 92.8%) for cervical cancer and 94.3% (92.6% to 95.7%) for CIN3 (table 3). The relative risk reduction estimates for the earlier period are not identical to those reported previously because we also had new data for the unvaccinated cohorts that affected the baseline rates.

**Table 3:** Estimated relative risk reductions (percentages) in incidence of invasive cervical cancer and CIN3 in the three cohorts offered HPV vaccination compared with the most recent unvaccinated cohort

Outcome by birth cohort	Relative risk reductions (95% CI)		
	Before 1 July 2019	From 1 July 2019	Combined (all data)
**Invasive cervical cancer**			
Cohort 5: Invited from age 24.5 and offered vaccine in school years 12-13 (ages 16-18)	32.5 (24.2 to 39.9)	47.2 (34.4 to 57.6)	35.5 (28.1 to 42.2)
Cohort 6: Invited from age 24.5 and offered vaccine in school years 10-11 (ages 14-16)	62.6 (51.8 to 71.0)	80.6 (71.7 to 86.6)	71.3 (64.3 to 76.9)
Cohort 7: Not invited before age 24.5 and offered vaccine in school year 8 (ages 12-13)	87.0 (72.5 to 93.9)	83.9 (63.8 to 92.8)	86.0 (75.5 to 92.0)
**CIN3**			
Cohort 5: Invited from age 24.5 and offered vaccine in school years 12-13 (ages 16-18)	37.9 (36.1 to 39.6)	36.2 (33.1 to 39.1)	37.7 (36.0 to 39.3)
Cohort 6: Invited from age 24.5 and offered vaccine in school years 10-11 (ages 14-16)	74.7 (73.0 to 76.3)	67.4 (65.4 to 69.4)	71.50 (70.1 to 72.8)
Cohort 7: Not invited before age 24.5 and offered vaccine in school year 8 (ages 12-13)	97.0 (96.0 to 97.7)	94.3 (92.6 to 95.7)	95.9 (95.0 to 96.6)

Estimates are adjusted for confounders and reported for periods 1 January 2006 to 30 June 2019, 1 July 2019 to 30 June 2020, and 1 January 2006 to 30 June 2020.

CI=confidence interval; CIN3=grade 3 cervical intraepithelial neoplasia; HPV=human papillomavirus.

Supplementary table S4 shows the full estimates from modelling the effects of vaccination in different levels of socioeconomic deprivation, with summary results reported in table 4, table 5, and table 6. The highest incidence rates for invasive cervical cancer were observed among women living in the most deprived areas (first fifth) but, while in the reference unvaccinated group there was a strong downward gradient moving from women in the most deprived areas to those in the least deprived, little difference was found between the second and fifth fifths of deprivation in the groups offered vaccination. In both the reference and the vaccination cohorts the highest rates of CIN3 occurred in those from the most deprived areas, but no clear trend was observed among the other four fifths of deprivation (see supplementary tables S5 and S6).

**Table 4:** Estimated number of invasive cervical cancers and CIN3s predicted and prevented by mid-2020 in the three cohorts of women offered HPV vaccination

	Predicted No of women with diagnosis* (95% CI)		No of cancers prevented*: scenarios A−B (95% CI)
	Scenario A: counterfactual	Scenario B: factual	
**Invasive cervical cancer**			
Index of multiple deprivation (fifths):			
1st (most deprived)	463 (424 to 501)	271 (238 to 304)	192 (141 to 242)
2nd	369 (338 to 399)	170 (144 to 196)	199 (158 to 239)
3rd	271 (248 to 294)	127 (105 to 149)	144 (112 to 176)
4th	212 (193 to 230)	120 (98 to 142)	92 (63 to 120)
5th (least deprived)	155 (142 to 169)	94 (75 to 113)	61 (38 to 85)
Total	1469 (1350 to 1589)	782 (727 to 837)	687 (556 to 819)
**CIN3**			
Index of multiple deprivation (fifths):			
1st (most deprived)	12 023 (11 761 to 12 285)	6902 (6693 to 7111)	5121 (4788 to 5455)
2nd	11 087 (10 845 to 11 328)	5314 (5138 to 5490)	5773 (5474 to 6071)
3rd	9341 (9135 to 9548)	4526 (4359 to 4693)	4815 (4551 to 5080)
4th	7918 (7741 to 8095)	3745 (3601 to 3889)	4173 (3945 to 4401)
5th (least deprived)	6292 (6150 to 6435)	2983 (2858 to 3108)	3309 (3120 to 3499)
Total	46 662 (45 697 to 47 627)	23 470 (23 097 to 23 843)	23 192 (22 163 to 24 220)

Results are reported under two scenarios: one as observed in the dataset (scenario B: factual) and one hypothetical where women had not been offered the HPV vaccination (scenario A: counterfactual).

CI=confidence interval; HPV=human papillomavirus.

* Numbers are rounded to nearest integers.

**Table 5:** Estimated cohort specific numbers of invasive cervical cancers predicted and prevented by mid-2020 among women in the least and most deprived areas

	Predicted No of invasive cervical cancers* (95% CI)				Predicted rates per 100 000 women years				% of cancers prevented: (A-B)/A
	Scenario A: counterfactual	Scenario B: factual	Difference: scenarios A-B†		Scenario A: counterfactual	Scenario B: factual	Difference: scenarios A-B		
**Cohort 5 (offered vaccine at age 16-18)**									
Index of multiple deprivation (fifths):									
1st (most deprived)	331 (304 to 358)	234 (205 to 263)	97 (58 to 137)		14.8	10.4	4.3		29.4
5th (least deprived)	107 (98 to 116)	80 (63 to 96)	27 (9 to 46)		8.5	6.3	2.2		25.6
**Cohort 6 (offered vaccine at age 14-16)**									
Index of multiple deprivation (fifths):									
1st (most deprived)	105 (94 to 115)	33 (26 to 40)	71 (59 to 84)		11.0	3.5	7.5		68.3
5th (least deprived)	38 (34 to 42)	13 (9 to 16)	25 (20 to 30)		6.5	2.2	4.3		66.8
**Cohort 7 (offered vaccine at age 12-13)**									
Index of multiple deprivation (fifths):									
1st (most deprived)	27 (24 to 31)	4 (2 to 7)	23 (19 to 27)		3.0	0.5	2.6		84.6
5th (least deprived)	10 (9 to 12)	2 (1 to 3)	9 (7 to 10)		1.8	0.3	1.5		83.5

Results are reported under two scenarios: one as observed in the dataset (scenario B: factual) and one hypothetical where women had not been offered the HPV vaccination (scenario A: counterfactual).

CI=confidence interval; HPV=human papillomavirus.

* Numbers rounded to nearest integers.

† Difference may not equal scenarios A−B due to rounding.

**Table 6:** Estimated cohort specific numbers of CIN3 predicted and prevented by mid-2020 among women in the least and most deprived areas

		Predicted No of women with diagnosis* (95% CI)				Predicted rates per 100 000 women years				% prevented: (A−B)/A
		Scenario A: counterfactual	Scenario B: factual	Difference: scenarios A-B†		Scenario A: counterfactual	Scenario B: factual	Difference: scenarios A−B		
**Cohort 5 (offered vaccine at age 16-18)**										
Index of multiple deprivation (fifths):										
1st (most deprived)		8396 (8223 to 8570)	5909 (5725 to 6093)	2487 (2235 to 2739)		375.3	264.1	111.2		29.6
5th (least deprived)		4224 (4134 to 4315)	2508 (2401 to 2615)	1716 (1577 to 1856)		334.3	198.5	135.8		40.6
**Cohort 6 (offered vaccine at age 14-16)**										
Index of multiple deprivation (fifths):										
1st (most deprived)		2981 (2901 to 3061)	963 (918 to 1008)	2019 (1927 to 2110)		314.6	101.6	213.0		67.7
5th (least deprived)		1689 (1642 to 1735)	460 (435 to 485)	1229 (1176 to 1282)		288.3	78.5	209.7		72.8
**Cohort 7 (offered vaccine at age 12-13)**										
Index of multiple deprivation (fifths):										
1st (most deprived)		646 (626 to 665)	30 (24 to 36)	615 (595 to 636)		72.0	3.4	68.6		95.3
5th (least deprived)		379 (368 to 391)	15 (12 to 18)	364 (352 to 377)		65.5	2.6	63.0		96.1

Results are reported under two scenarios: one as observed in the dataset (scenario B: factual) and one hypothetical where women had not been offered the HPV vaccination (scenario A: counterfactual).

CI=confidence interval; HPV=human papillomavirus.

* Numbers rounded to nearest integers.

† Difference may not equal scenarios A-B due to rounding.

Overall, our model estimated that 687 (95% CI 556 to 819) cervical cancers and 23 192 (22 163 to 24 220) CIN3s had been prevented by the vaccination programme up to mid-2020 among young women in England (table 4). The greatest numbers for cervical cancer were prevented in women in the most deprived areas (192 and 199 for first and second fifths, respectively) and the fewest in women in the least deprived fifth (61 cancers prevented). The number of women with CIN3 prevented was high across all deprivation groups but greatest among women living in the more deprived areas: 5121 and 5773 for first and second fifths, respectively, compared with 4173 and 3309 in the fourth and fifth fifths, respectively. When we looked at the corresponding cohort specific figures (table 5 and table 6), we noticed differences between the cohorts, particularly for CIN3. In all three cohorts offered vaccination the numbers and rates of prevented cervical cancers were much higher in women from the most deprived areas than least deprived areas (table 5). The proportion of women with prevented cervical cancer in each cohort was, however, similar between the first and fifth fifths of deprivation. For CIN3 (table 6), the results were more complicated. In women offered vaccination at age 16-18 years (cohort 5), the proportion of cervical cancers prevented was substantially less in those from the most deprived areas (29.6%) compared with those from the least deprived areas (40.6%). An inequality still existed in cohorts 6 and 7, but it was greatly reduced (67.7% *v* 72.8% in cohort 6 and 95.3% *v* 96.1% in cohort 7).

## Discussion

In England, the social-class gradient for cervical cancer is one of the steepest of any cancers: women in the most deprived fifth have had double the risk of those in the least deprived fifth.^39^ ^40^ Some of this results from differences in exposure to HPV and risk of an infection becoming persistent,^41^ but differential uptake of cervical screening has also been an important factor. Previous research has highlighted the need for new engagement strategies to improve attendance for cervical screening among young women living in more socially deprived areas.^42^ Encouragingly, the coverage of HPV vaccination has been (at least for the routine campaign and before the covid-19 pandemic) uniformly high.^43^ It is, however, important to investigate whether immunisation—including the indirect effects achieved by high uptake—is helping to reduce health inequalities.

Using population based cancer registrations updated to mid-2020, which provided information on about twice the expected number of cancers in women offered HPV vaccination aged 12-13 years than in our previous analysis, we were able to show that the high vaccination effectiveness seen previously was confirmed with more recent data. The largest differences between the old and the new data were found for cohort 6 (the catch-up group offered the vaccine at age 14-16 years): for cervical cancer the estimated effectiveness increased, whereas for CIN3 it decreased. The reasons behind these differences are unclear. The results for cohorts 6 and 7 in the new data are more in keeping with what we would have expected given that the proportion of disease caused by HPV types 16 and 18 is greater for invasive cancer than for CIN3.

We also investigated the effect of the HPV immunisation programme by socioeconomic deprivation. Overall, we found that the programme was associated with a substantial reduction in the expected number of women with cervical cancers and CIN3 in all fifths of deprivation. For cervical cancer before vaccination, the downward gradient with decreasing deprivation was strong. In all cohorts offered vaccination, the highest rate was still seen among women living in the most deprived areas, but little difference was observed between women living in the second to fifth deprived areas. For CIN3, similar patterns were observed for the reference unvaccinated group and the three cohorts offered vaccination, but rates were greatly reduced in all fifths of deprivation in the latter. When we compared women in the most deprived areas with those in the least deprived areas in terms of percentage of disease averted, we observed differences across the cohorts for CIN3, with women in the least deprived areas in the older catch-up cohort (vaccine offered at age 16-18 years) having a greater proportion of averted CIN3s after HPV immunisation than women in the most deprived area (40.6% *v* 29.6%). The same, although to a much less extent, was observed for the younger catch-up cohort (72.8% *v* 67.7%). For invasive cervical cancer, we found no evidence of a less beneficial impact (in terms of percentage of cases averted) of the vaccination in women living in the most deprived areas; in fact, especially for the older catch-up cohort, the percentage was slightly higher in women in the most deprived areas compared with those in the least deprived areas.

The observed incidences of cervical cancer and CIN3 depend on three key factors: the intensity of exposure to HPV infections (including age at first exposure), the uptake of cervical screening, and HPV vaccination coverage. It is therefore difficult to disentangle the effects of these three drivers on the index of multiple deprivation specific rates with the data at hand. The health inequality in CIN3 in cohort 5 might result from the lower vaccination coverage among women in the most deprived areas since at age 16-18 years when they became eligible for vaccination more of those from the most deprived fifth may not have been in school or, for other reasons, may have missed the offer of HPV immunisation. These observations are consistent with previous understanding that higher uptake of catch-up vaccination was associated, although not as strongly as in some countries, with lower deprivation.^25^ It is, however, reassuring that cohorts 6 and 7 showed little inequality in relative reductions in cancer (as in vaccination coverage).

However, since the UK has recently announced a change to a one dose schedule for routine HPV vaccination, ensuring this change achieves high coverage (including in the birth cohorts currently with lower coverage owing to covid-19 related interruption to schooling, and to immunisation services) is important to maintain the effects we have seen on cervical disease and on inequalities. Further investigations could be carried out in the future to check for any effect on cancer incidence caused by covid-19, gender neutral vaccination (since 2019), a change in the type of vaccine used, or reduced dose schedules.

### Strengths and limitations of this study

Our analysis has several strengths. Our study provides direct evidence for the effect of a public health intervention (such as HPV vaccination) on cancer rates by deprivation. We used high quality data from population based cancer registries and were able to investigate the extent of socioeconomic inequalities in cohorts offered vaccination and whether the effectiveness of the HPV immunisation continued in an additional year of follow-up. The code for the analysis was written and tested using simulated data and an independent analyst later ran the code on the real dataset, guaranteeing reliable and robust results and preserving patient confidentiality.

The main limitations of our study are that it was observational and individual level data on vaccination status were not available. However, previous published research^14^ provided detailed information on potential confounding factors and the best way to adjust for these in the analysis. Additionally, the discontinuities in vaccine uptake with date of birth makes this study powerful and less prone to biases from unobserved confounders than an analysis based on individual level data on HPV vaccination status.

Women born after 1 September 1999 were offered the Gardasil vaccine from 1 September 2012. As these women were at most aged 20 years and 10 months at the end of the study follow-up (30 June 2020), it is not yet possible with the data available to compare the effectiveness of the programme among those offered Cervarix and those offered Gardasil. This additional comparative analysis will become feasible with a longer follow-up on the recipients of Gardasil.

### Policy implications

We found that the high effectiveness of the national HPV immunisation continued in the additional year of follow-up (July 2019 to June 2020). This is encouraging as it validates the previously published results and further supports consideration of more limited cervical screening for cohorts with high vaccination coverage aged 12-13 years. Moreover, although women living in the most deprived areas are still at higher risk of cervical cancer than those in less deprived areas, the HPV vaccination programme is associated with substantially lowered rates of disease across all fifths of socioeconomic deprivation. For cervical cancer, this has led to the levelling-up of the rates across the second to fifth fifths of deprivation so that the strong downward gradient observed in the reference unvaccinated cohort is no longer present in the cohorts offered vaccination. For CIN3, in the older catch-up cohorts women living in the least deprived areas seem to have benefited more from vaccination than those living in the most deprived areas, but the rates were still greatly reduced in all socioeconomic groups. Cervical screening strategies for women offered vaccination should carefully consider the differential effect both on rates of disease and on inequalities that are evident among women offered catch-up vaccination.

### Conclusions

The HPV vaccination programme in England has not only been associated with a substantial reduction in incidence of cervical neoplasia in targeted cohorts, but also in all socioeconomic groups. This shows that well planned and executed public health interventions can both improve health and reduce health inequalities.

**What is already known on this topic**

In England, immunisation against human papillomavirus (HPV) has been associated with greatly reduced incidence rates of cervical cancer and grade 3 cervical intraepithelial neoplasia (CIN3) up to June 2019, especially among women offered routine vaccination at age 12-13 years
The social-class gradient for cervical cancer incidence has been one of the steepest of any cancers
Concern has been raised that HPV vaccination could least benefit those at highest risk of cervical cancer

**What this study adds**

The high effectiveness of vaccination against HPV seen previously continued during an additional year of follow-up, from July 2019 to June 2020
The English HPV vaccination programme was associated with substantially lower rates of cervical cancer and CIN3 in all fifths of socioeconomic deprivation, although the highest rates remained among women in the most deprived areas
For cervical cancer, the strong downward gradient from high to low deprivation observed in the reference unvaccinated cohort was no longer present among those offered vaccination

## Data Availability

The cancer registry data analysed for this paper are securely held by the National Disease Registration Service (NDRS). Requests to access the data can be made through NHS England’s DARS service (https://digital.nhs.uk/services/data-access-request-service-dars). The Simulacrum (https://simulacrum.healthdatainsight.org.uk/) is a synthetic dataset developed by Health Data Insight and derived from anonymous cancer data provided by NHS England’s NDRS. Mid-year population estimates are freely downloadable from the Office for National Statistics website (https://www.ons.gov.uk/).

## Web extra

Extra material supplied by authors

Supplementary information: Additional tables S1-S6, figure S1, and details on index of multiple deprivation and calculation of the population estimates

## Notes

Contributors: PS had the original idea. He is the guarantor. MF and PS conceptualised the study and prepared the study protocol, which was subsequently reviewed by the other co-authors. MF wrote and tested the Stata code (checked by PS) for the data analysis and drafted the manuscript. BN extracted the dataset and ran the Stata code on it. All authors critically reviewed and approved the final submitted version. The corresponding author attests that all listed authors meet authorship criteria and that no others meeting the criteria have been omitted.

Transparency: The lead author (the manuscript’s guarantor) affirms that the manuscript is an honest, accurate, and transparent account of the study being reported; that no important aspects of the study have been omitted; and that any discrepancies from the study as planned (and, if relevant, registered) have been explained.

Dissemination to participants and related patient and public communities: The results of this research will be disseminated through the media, blogs and scientific meetings and will inform the design and implementation of interventions to reduce health inequalities. We will also work with others to produce information for the public to support human papillomavirus immunisation and cervical screening programmes and, if the opportunity arises, to contribute summary data for an international meta-analysis of similar studies.

Provenance and peer review: Not commissioned; externally peer reviewed.
