# Supplementary material for: Effect of the HPV vaccination programme on incidence of cervical cancer and grade 3 cervical intraepithelial neoplasia by socioeconomic deprivation in England: population based observational study
Source: BMJ. 2024 May 15;385:e077341. doi: 10.1136/bmj-2023-077341 (PMC11094700; doi:10.1136/bmj-2023-077341)
Supplement: Supplementary file 1 — Supplementary information: Additional tables S1-S6, figure S1, and details on index of multiple deprivation and calculation of the population estimates [file falm077341.ww.pdf]

Supplementary material

Table S1: Main effects and interaction terms included in our core model. To validate previous analyses of the effectiveness of the HPV vaccination programme, we split the main effect of each "vaccinated" cohort into 2 sub-group effects depending on whether the data related to 1 January 2006 - 30 June 2019 or to 1 July 2019 - 30 June 2020 (this corresponded to adding three cohort-by-period interaction terms). By contrast, to investigate the effectiveness by socio-economic deprivation, we added binary variables for the IMD quintiles (main effects) and deprivation-by-cohort interactions.

Effects	Description	Variable type	Coding and additional information
**Main effects**			
Age (A)	age at diagnosis	categorical (7 age groups)	cut-points at ages 20, 24.5, 26, 30, 35, 45 and 55 years
Period (P):			
Drift	linear time trend	continuous (in months)	centered at January 2016
Seasonality	seasonal changes in diagnosis	categorical (4 seasons)	Jan - Mar, Apr - Jun, Jul - Sep and Oct - Dec
Under-registration and COVID-19	possible under-registration and COVID-19 disruptions in recent years of diagnosis	categorical (4 intervals)	Jan 2006 - Dec 2018, Jan 2019 - Sep 2019, Oct 2019 - Mar 2020 and Apr 2020 - Jun 2020
Cohort (C)	birth cohorts	categorical (7 groups)	see Falcaro et al. (2021)[a] and Figure 1
**Age-by-period interactions**			
Jade Goody effect	variation in cervical screening rates following the diagnosis and death of the TV celebrity Jade Goody	binary	1 for age 20 to <50 years and diagnosis in Jan-Jun 2009, 0 otherwise
Awareness campaign	expected increase in incidence due to the cervical screening awareness campaign launched in March 2019 by PHE	binary	1 for age≥24.5 years and diagnosis in Mar-Jun 2019, 0 otherwise
**Age-by-cohort interactions**			
Changes in 1st screening invitation	increased diagnosis of prevalent cancer cases arising from policy changes in the age of first invitation to screening	6 binary variables	See Falcaro et al. (2021)[a]

[a] Falcaro et al. (2021). The effects of the national HPV vaccination programme in England, UK, on cervical cancer and grade 3 cervical intraepithelial neoplasia incidence: a register-based observational study. *Lancet* 398(10316): 2084-2092.

Table S2: Number of diagnoses and women-years by birth cohort and age group. Figures are reported separately for the full data set and the last 12 months of the study follow-up.

	All data			Additional data since previous publication		
	Cervical cancer	CIN3	Women-years (millions)	Cervical cancer	CIN3	Women-years (millions)
**Birth cohort**						
**1**: invited from age 20 and unvaccinated	25,062	211,501	186.3	1,397	6,210	10.8
**2**: invited from age 20 or 25 and unvaccinated	1,021	21,629	6.2	91	906	0.5
**3**: invited from age 25 and unvaccinated	2,453	59,881	16.5	303	3,778	1.3
**4**: invited from age 24.5 and unvaccinated	650	18,747	5.1	87	1,468	0.5
**5**: invited from age 24.5 and offered vaccine in school years 12-13	669	19,920	9.1	108	2,961	1.1
**6**: invited from age 24.5 and offered vaccine in school years 10-11	100	3,441	4	30	1,787	0.7
**7**: not invited before age 24.5 and offered vaccine in school year 8	13	109	3.9	6	60	1.4
**Age at diagnosis (in years)**						
20 to <24.5	337	9,954	22.3	8	68	1.4
24.5 to <26	1,609	59,539	8.1	30	1,601	0.6
26 to <30	3,533	92,568	21.2	173	3,962	1.5
30 to <65	24,489	173,167	179.5	1,811	11,539	12.8

Table S3: Estimated incidence rate ratios (IRRs) along with their p-values and 95% confidence intervals (CIs) from the analysis we performed to evaluate if the high effectiveness of the vaccination previously reported has continued for diagnoses in July 2019 - June 2020.

	Invasive cervical cancer				CIN3			
	IRR	p	95% CI		IRR	p	95% CI	
**Age**								
20 to <24.5	1.000				1.000			
24.5 to <26	6.294	<0.001	5.438	7.284	7.437	<0.001	7.195	7.686
26 to <30	8.062	<0.001	7.147	9.093	6.916	<0.001	6.726	7.110
30 to <35	8.784	<0.001	7.752	9.953	4.341	<0.001	4.210	4.475
35 to <45	7.840	<0.001	6.902	8.906	1.994	<0.001	1.931	2.058
45 to <55	5.289	<0.001	4.651	6.015	0.627	<0.001	0.606	0.649
55 to <65	4.676	<0.001	4.108	5.322	0.259	<0.001	0.250	0.270
**Drift**	1.008	<0.001	1.004	1.011	0.992	<0.001	0.991	0.993
**Seasonality**								
Jan-Mar	1.000				1.000			
Apr-June	1.014	0.392	0.982	1.048	1.046	<0.001	1.035	1.057
July-Sept	1.012	0.471	0.979	1.046	1.046	<0.001	1.034	1.057
Oct-Dec	1.030	0.083	0.996	1.064	1.043	<0.001	1.032	1.055
**Under-registration and COVID19**								
Jan 2006 - Dec 2018	1.000				1.000			
Jan 2019 - Sep 2019	1.048	0.202	0.975	1.127	1.128	<0.001	1.100	1.157
Oct 2019 - Mar 2020	1.008	0.816	0.940	1.082	1.026	0.059	0.999	1.054
Apr 2020 - Jun 2020	0.799	<0.001	0.720	0.886	0.680	<0.001	0.652	0.709
**Awareness campaign**	1.137	0.011	1.030	1.256	1.111	<0.001	1.073	1.150
**Jade Goody effect**	1.319	<0.001	1.234	1.410	1.411	<0.001	1.384	1.438
**Age-by-cohort interactions**								
CxA_25y_mixed_1	3.318	<0.001	2.632	4.182	2.970	<0.001	2.819	3.129
CxA_25y_mixed_2	1.809	<0.001	1.388	2.357	1.736	<0.001	1.648	1.829
CxA_25y_1	3.536	<0.001	3.023	4.134	3.881	<0.001	3.753	4.013
CxA_25y_2	1.798	<0.001	1.506	2.146	1.803	<0.001	1.740	1.868
CxA_24y5_1	2.464	<0.001	2.079	2.920	2.898	<0.001	2.777	3.024
CxA_24y5_2	2.566	<0.001	2.154	3.056	2.666	<0.001	2.572	2.763
**Birth cohort and before/from 1 July 2019**								
1	1.002	0.975	0.909	1.103	0.980	0.099	0.957	1.004
2	1.094	0.111	0.980	1.222	1.029	0.034	1.002	1.056
3	1.064	0.208	0.966	1.172	1.030	0.011	1.007	1.053
4	1.000				1.000			
5 & before 1 July 2019	0.675	<0.001	0.601	0.758	0.621	<0.001	0.604	0.639
5 & from 1 July 2019	0.528	<0.001	0.424	0.656	0.638	<0.001	0.609	0.669
6 & before 1 July 2019	0.374	<0.001	0.290	0.482	0.253	<0.001	0.237	0.270
6 & from 1 July 2019	0.194	<0.001	0.134	0.283	0.326	<0.001	0.306	0.346
7 & before 1 July 2019	0.130	<0.001	0.061	0.275	0.030	<0.001	0.023	0.040
7 & from 1 July 2019	0.161	<0.001	0.072	0.362	0.057	<0.001	0.043	0.074
**_cons**	2.11E-05	<0.001	1.85E-05	2.41E-05	6.15E-04	<0.001	5.96E-04	6.33E-04

Table S4: Estimated incidence rate ratios (IRRs) along with their p-values and 95% confidence intervals (CIs) from the model with adjustment for IMD quintile.

	Invasive cervical cancer				CIN3			
	IRR	p	95% CI		IRR	p	95% CI	
**Age in years**								
20 to <24.5	1.000				1.000			
24.5 to <26	6.124	<0.001	5.299	7.077	7.495	<0.001	7.253	7.745
26 to <30	7.841	<0.001	6.971	8.820	6.974	<0.001	6.786	7.167
30 to <35	8.715	<0.001	7.711	9.851	4.382	<0.001	4.252	4.517
35 to <45	7.980	<0.001	7.042	9.042	2.019	<0.001	1.956	2.084
45 to <55	5.457	<0.001	4.810	6.191	0.636	<0.001	0.615	0.658
55 to <65	4.871	<0.001	4.289	5.532	0.263	<0.001	0.253	0.274
**Drift**	1.007	<0.001	1.004	1.011	0.992	<0.001	0.990	0.993
**Seasonality**								
Jan-Mar	1.000				1.000			
Apr-June	1.014	0.407	0.981	1.048	1.046	<0.001	1.035	1.058
July-Sept	1.011	0.510	0.978	1.045	1.047	<0.001	1.035	1.058
Oct-Dec	1.030	0.082	0.996	1.064	1.043	<0.001	1.032	1.055
**Under-registration and COVID19**								
Jan 2006 - Dec 2018	1.000				1.000			
Jan 2019 - Sep 2019	1.039	0.297	0.967	1.117	1.138	<0.001	1.110	1.167
Oct 2019 - Mar 2020	0.987	0.720	0.922	1.058	1.049	<0.001	1.023	1.075
Apr 2020 - Jun 2020	0.783	<0.001	0.706	0.868	0.695	<0.001	0.667	0.724
**Awareness campaign**	1.155	0.004	1.047	1.275	1.094	<0.001	1.057	1.132
**Jade Goody effect**	1.318	<0.001	1.233	1.409	1.411	<0.001	1.384	1.438
**Age-by-cohort interactions**								
CxA_25y_mixed_1	3.309	<0.001	2.627	4.167	2.966	<0.001	2.816	3.125
CxA_25y_mixed_2	1.804	<0.001	1.385	2.349	1.734	<0.001	1.646	1.827
CxA_25y_1	3.529	<0.001	3.017	4.128	3.879	<0.001	3.752	4.011
CxA_25y_2	1.797	<0.001	1.505	2.146	1.803	<0.001	1.740	1.868
CxA_24y5_1	2.559	<0.001	2.164	3.026	2.894	<0.001	2.775	3.017
CxA_24y5_2	2.619	<0.001	2.204	3.113	2.669	<0.001	2.577	2.764
**Birth cohort (C)**								
1: invited from age 20 and unvaccinated	1.001	0.989	0.908	1.102	0.978	0.073	0.955	1.002
2: invited from age 20 or 25 and unvaccinated	1.092	0.119	0.978	1.219	1.027	0.043	1.001	1.055
3: invited from age 25 and unvaccinated	1.065	0.201	0.967	1.173	1.029	0.013	1.006	1.052
4: invited from age 24.5 and unvaccinated	1.000				1.000			
5: invited from age 24.5 and offered vaccine in school years 12-13	0.706	<0.001	0.609	0.818	0.704	<0.001	0.678	0.730
6: invited from age 24.5 and offered vaccine in school years 10-11	0.317	<0.001	0.250	0.403	0.323	<0.001	0.306	0.341
7: not invited before age 24.5 and offered vaccine in school year 8	0.156	<0.001	0.089	0.274	0.047	<0.001	0.038	0.057
**IMD quintile**								
1	1.000				1.000			
2	0.789	<0.001	0.763	0.816	0.912	<0.001	0.902	0.923
3	0.700	<0.001	0.676	0.725	0.929	<0.001	0.918	0.940
4	0.651	<0.001	0.628	0.674	0.937	<0.001	0.925	0.948
5	0.583	<0.001	0.562	0.606	0.908	<0.001	0.897	0.920
**IMD*C interactions**								
2#vaccinated	0.794	0.022	0.652	0.967	0.843	<0.001	0.805	0.883
3#vaccinated	0.811	0.054	0.655	1.004	0.856	<0.001	0.815	0.900
4#vaccinated	0.981	0.863	0.788	1.221	0.836	<0.001	0.795	0.879
5#vaccinated	1.054	0.669	0.829	1.340	0.844	<0.001	0.800	0.890
**_cons**	2.79E-05	<0.001	2.44E-05	3.19E-05	6.47E-04	<0.001	6.28E-04	6.67E-04

Table S5: Adjusted IRRs for invasive cervical cancer and CIN3 with a common reference group (cohort 4 and IMD 1). Figures between brackets represent 95% CIs.

	**Birth cohort**			
	**4**	**5**	**6**	**7**
**Invasive cervical cancer**				
IMD quintile				
1 - most deprived	**1.000**	**0.706**<br>(0.609, 0.818)	**0.317**<br>(0.250, 0.403)	**0.156**<br>(0.089, 0.274)
2	**0.789**<br>(0.763, 0.816)	**0.442**<br>(0.372, 0.527)	**0.199**<br>(0.153, 0.258)	**0.098**<br>(0.055, 0.174)
3	**0.700**<br>(0.676, 0.725)	**0.400**<br>(0.330, 0.486)	**0.180**<br>(0.137, 0.236)	**0.088**<br>(0.049, 0.157)
4	**0.651**<br>(0.628, 0.674)	**0.450**<br>(0.369, 0.550)	**0.202**<br>(0.154, 0.266)	**0.099**<br>(0.056, 0.177)
5 - least deprived	**0.583**<br>(0.562, 0.606)	**0.434**<br>(0.348, 0.541)	**0.195**<br>(0.146, 0.261)	**0.096**<br>(0.053, 0.173)
**CIN3**				
IMD quintile				
1 - most deprived	**1.000**	**0.704**<br>(0.678, 0.730)	**0.323**<br>(0.306, 0.341)	**0.047**<br>(0.038, 0.057)
2	**0.912**<br>(0.902, 0.923)	**0.541**<br>(0.520, 0.563)	**0.248**<br>(0.235, 0.262)	**0.036**<br>(0.029, 0.044)
3	**0.929**<br>(0.918, 0.940)	**0.560**<br>(0.537, 0.584)	**0.257**<br>(0.242, 0.272)	**0.037**<br>(0.030, 0.045)
4	**0.937**<br>(0.925, 0.948)	**0.551**<br>(0.527, 0.576)	**0.253**<br>(0.238, 0.268)	**0.037**<br>(0.030, 0.045)
5 - least deprived	**0.908**<br>(0.897, 0.920)	**0.539**<br>(0.514, 0.565)	**0.247**<br>(0.233, 0.263)	**0.036**<br>(0.029, 0.044)

Table S6: Adjusted IRRs for invasive cervical cancer and CIN3 with a separate reference group (IMD 1) within each cohort. Figures between brackets represent 95% CIs.

	**Invasive cervical cancer**		**CIN3**	
	**Cohort 4**	**"Vaccinated" cohorts (cohorts 5 to 7)**	**Cohort 4**	**"Vaccinated" cohorts (cohorts 5 to 7)**
**IMD quintile**				
1 - most deprived	**1.000**	**1.000**	**1.000**	**1.000**
2	**0.789** (0.763, 0.816)	**0.627** (0.516, 0.761)	**0.912** (0.902, 0.923)	**0.769** (0.735, 0.804)
3	**0.700** (0.676, 0.725)	**0.567** (0.459, 0.701)	**0.929** (0.918, 0.940)	**0.796** (0.759, 0.835)
4	**0.651** (0.628, 0.674)	**0.638** (0.514, 0.792)	**0.937** (0.925, 0.948)	**0.783** (0.746, 0.822)
5 - least deprived	**0.583** (0.562, 0.606)	**0.615** (0.485, 0.779)	**0.908** (0.897, 0.920)	**0.766** (0.727, 0.807)

Figure S1: Cervical screening coverage in England between 2009/2010 and 2021/2022 by IMD quintile for women aged 25 to 49 years. Data were collected by the NHS Cervical Screening Programme (downloadable from https://fingertips.phe.org.uk/, indicator id = 93725).

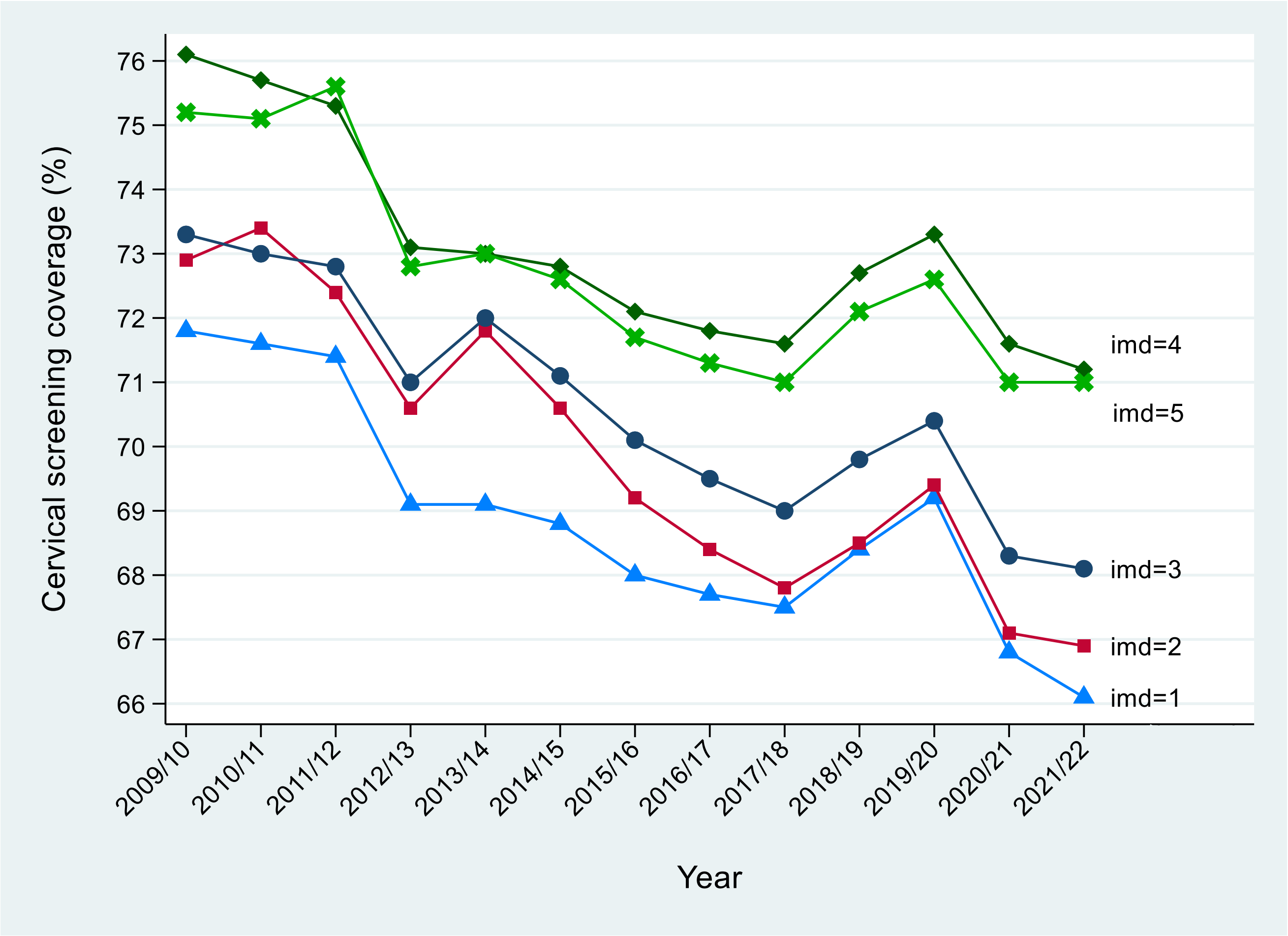

IMD versions used in the ONS's population estimates and in the NDRS's cancer registration data

- **ONS data**

IMD version	Population data
2007	2006 to 2008
2010	2009 to 2012
2015	2013 to 2017
2019	2018 to 2020

- **NDRS data**

IMD version	Year of diagnosis
2007	2006
2010	2007 to 2009
2015	2010 to 2013
2019	2014 to 2020

Calculation of the population estimates required in our statistical analysis

- Mid-year population estimates for England by IMD decile, sex and single year of age were retrieved from the following ONS's web pages:

  - years 2001 to 2018
  https://www.ons.gov.uk/peoplepopulationandcommunity/birthsdeathsandmarriages/deaths/adhocs/11169deathregistrationsandpopulationsbyindexofmultipledeprivationengland2001to2018

  - year 2019
  https://www.ons.gov.uk/peoplepopulationandcommunity/birthsdeathsandmarriages/deaths/adhocs/12413deathregistrationsandpopulationsbyindexofmultipledeprivationimddecileenglandandwales2019

  - year 2020
  https://www.ons.gov.uk/peoplepopulationandcommunity/populationandmigration/populationestimates/adhocs/13773populationsbyindexofmultipledeprivationimddecileenglandandwales2020

- We started by creating a data set containing the mid-year female population estimates by IMD decile, single year of age (20 to 64) and calendar year (2006 to 2020).

year	IMD_decile	Pop20	Pop21	Pop22	…	Pop63	Pop64
2006	1	44,994	46,071	45,516	…	19,981	18,732
2006	2	43,488	44,401	43,884	…	20,651	19,299
2006	3	40,658	41,275	40,721	…	22,502	20,797
2006	4	37,321	38,407	37,234	…	24,683	22,656
2006	5	34,526	34,558	33,833	…	27,684	25,324
2006	6	31,421	31,998	31,182	…	28,954	26,068
2006	7	27,697	28,066	27,689	…	30,964	27,976
2006	8	24,817	25,644	25,757	…	30,828	27,548
2006	9	23,900	23,945	24,221	…	31,593	27,459
2006	10	20,446	20,765	22,140	…	30,793	26,734
…							
2020	1	35,675	36,419	36,419	…	26,231	24,646
2020	2	36,325	38,068	38,409	…	27,289	25,887
2020	3	36,820	38,707	40,439	…	28,661	27,287
2020	4	37,039	39,042	39,854	…	31,146	30,035
2020	5	34,285	35,543	35,634	…	32,191	31,093
2020	6	32,603	33,808	34,808	…	33,886	32,797
2020	7	28,149	29,771	30,716	…	34,368	33,036
2020	8	26,031	27,682	28,574	…	34,430	33,182
2020	9	23,578	25,024	26,281	…	33,736	32,892
2020	10	24,991	24,503	24,662	…	33,723	32,452

- The data set was then collapsed and reshaped in long format so to have 1 record for each unique combination of values for IMD quintile, age (A) and period (P). The resulting data set looked as follows:

A	P	imd	Pop
20	2006	1	88,482
20	2007	1	89,401
20	2008	1	90,776
20	2009	1	83,545
20	2010	1	84,468
20	2011	1	88,008
20	2012	1	87,657
20	2013	1	81,990
20	2014	1	80,309
20	2015	1	78,311
20	2016	1	79,228
20	2017	1	80,568
20	2018	1	74,437
20	2019	1	74,074
20	2020	1	72,000
21	2006	1	90,472
21	2007	1	91,463
21	2008	1	92,870

...			
64	2015	5	66,610
64	2016	5	64,950
64	2017	5	65,671
64	2018	5	65,264
64	2019	5	64,299
64	2020	5	65,344

- To derive the values for the birth cohort (C), we had to take into account that for each combination of A and P (each recorded in integer years) there are 2 possible years of birth. For example, women who were aged 30 years old on 1 May 2015 were born either in 1985 or in 1984 depending on whether they had already celebrated their birthday that year (e.g. date of birth could be 5 Apr 1985 or 5 June 1984).
- We therefore split the population figures into monthly intervals for calendar time and date of birth and we derived the corresponding year of birth as C=P-A if month of birth < calendar month and P-A-1 if month of birth > calendar month. The population figures related to records with month of birth = calendar month were further split into equal parts between C=P-A and C=P-A-1.
